# Core Outcome Domains for early phase clinical trials of sound-, psychology-, and pharmacology-based interventions to manage chronic subjective tinnitus in adults: the COMIT’ID study protocol for using a Delphi process and face-to-face meetings to establish consensus

**DOI:** 10.1186/s13063-017-2123-0

**Published:** 2017-08-23

**Authors:** Kathryn Fackrell, Harriet Smith, Veronica Colley, Brian Thacker, Adele Horobin, Haúla F. Haider, Alain Londero, Birgit Mazurek, Deborah A. Hall

**Affiliations:** 1 NIHR Nottingham Hearing Biomedical Research Centre, Ropewalk House, 113 The Ropewalk, Nottingham, NG1 5DU UK; 2 Otology and Hearing Group, Division of Clinical Neuroscience, School of Medicine, University of Nottingham, Nottingham, NG7 2UH UK; 3 Nottingham University Hospitals NHS Trust, Queen’s Medical Centre, Derby Road, Nottingham, NG7 2UH UK; 4 ENT Department of Hospital Cuf Infante Santo, Nova Medical School, Travessa do Castro, 3, 1350-070 Lisbon, Portugal; 5 Service ORL et CCF, Consultation Acouphène et Hyperacousie, Hôpital Européen G. Pompidou, 20, rue Leblanc, 75015 Paris, France; 6 Tinnitus Center, Charite University Hospital, Chariteplatz 1, 10117 Berlin, Germany

**Keywords:** Consensus methods, Core outcome set, Delphi process, Drugs

## Abstract

**Background:**

The reporting of outcomes in clinical trials of subjective tinnitus indicates that many different tinnitus-related complaints are of interest to investigators, from perceptual attributes of the sound (e.g. loudness) to psychosocial impacts (e.g. quality of life). Even when considering one type of intervention strategy for subjective tinnitus, there is no agreement about what is critically important for deciding whether a treatment is effective. The main purpose of this observational study is, therefore to, develop Core Outcome Domain Sets for the three different intervention strategies (sound, psychological, and pharmacological) for adults with chronic subjective tinnitus that should be measured and reported in every clinical trial of these interventions. Secondary objectives are to identify the strengths and limitations of our study design for recruiting and reducing attrition of participants, and to explore uptake of the core outcomes.

**Methods:**

The ‘Core Outcome Measures in Tinnitus: International Delphi’ (COMIT’ID) study will use a mixed-methods approach that incorporates input from health care users at the pre-Delphi stage, a modified three-round Delphi survey and final consensus meetings (one for each intervention). The meetings will generate recommendations by stakeholder representatives on agreed Core Outcome Domain Sets specific to each intervention. A subsequent step will establish a common cross-cutting Core Outcome Domain Set by identifying the common outcome domains included in all three intervention-specific Core Outcome Domain Sets. To address the secondary objectives, we will gather feedback from participants about their experience of taking part in the Delphi process. We aspire to conduct an observational cohort study to evaluate uptake of the core outcomes in published studies at 7 years following Core Outcome Set publication.

**Discussion:**

The COMIT’ID study aims to develop a Core Outcome Domain Set that is agreed as critically important for deciding whether a treatment for subjective tinnitus is effective. Such a recommendation would help to standardise future clinical trials worldwide and so we will determine if participation increases use of the Core Outcome Set in the long term.

**Trial registration:**

This project has been registered (November 2014) in the database of the Core Outcome Measures in Effectiveness Trials (COMET) initiative.

**Electronic supplementary material:**

The online version of this article (doi:10.1186/s13063-017-2123-0) contains supplementary material, which is available to authorized users.

## Background

Tinnitus is the sensation of noise ﻿such as ringing, buzzing or hissing sound perceived in the ears or head. Over 70 million people in Europe and more than 50 million people in the USA experience the condition. In few cases, tinnitus is objective with vascular or muscular causes. Most tinnitus cases are subjective, which means that the sounds are perceived only by the patient, has no clinically identifiable source of the sound and whose pathophysiology is not fully understood. This project is limited to subjective tinnitus, and so in the remainder of the article wherever we use the term ‘tinnitus’ we are referring to subjective tinnitus.

Tinnitus is a symptom that can be chronic and disabling for some. However, it is a complex condition that is challenging to manage because it is associated with a diverse range of patient complaints, including perceived loudness, sleep problems, difficulties in listening and concentration, effects on psychological wellbeing, daily life and on general health [1–3]. It may also have negative effects on personal quality of life, as well as a societal impact in terms of social withdrawal and impaired work performance [4, 5]. Each of these patient complaints could be defined as a domain (i.e. a distinct element) of tinnitus which could determine how to measure whether a treatment has worked. The process of measuring tinnitus is made all the more challenging since individual patients tend to report different profiles of tinnitus-related complaint domains. Moreover, because treatment options remain palliative rather than curative any judgements about therapeutic benefit typically concern a relative improvement and not simply a binary ‘yes/no’ or ‘present/absent’ decision.

At present, there is no consensus on what are the critically important domains of tinnitus that should be assessed to determine whether the responses to treatment are beneficial or not for patients. Our recent systematic review examining 228 published clinical trials for tinnitus, identified 35 primary domain outcomes and 60 secondary domains’ outcomes spanning nine broad categories (tinnitus percept, impact of tinnitus, co-occurring complaints, health-related quality of life, body structures and function, adverse events or harms, treatment satisfaction, treatment-related outcomes and ‘unclear or not specified’) [6]. Inconsistent outcome reporting can severely hinder identifying and interpreting the relative merits of the various interventions or therapeutic approaches that are currently on offer or under investigation [7, 8]. If all clinical trials for tinnitus used and reported results for the same set of agreed outcomes, they could be compared and combined. This would make it much easier to make sense of all the knowledge produced and reduce unnecessary research waste. The main goal, therefore, is to advance the standards for clinical trials of interventions for tinnitus by developing an agreed list of outcome domains that are critically important for determining whether a treatment has worked.

Our systematic review of 228 published clinical trials also identified at least eight broad classes of intervention strategies that have been assessed in clinical trials [6]. Each of these intervention strategies has a different targeted rationale, and is focussed on alleviating different outcome domains of tinnitus. For example, sound-based interventions, such as hearing aids and sound generators, intend to reduce loudness (e.g. [8]), whilst psychologically informed interventions are intended to improve mental health and wellbeing (e.g. [9]). Therefore, any agreed list of outcome domains is likely to be more appropriate for one intervention strategy than another. This review [6] supported an informed choice about what are the major intervention strategies to be considered in the present study. Intervention strategies most commonly reported from 2006 to 2015 were pharmacological (*n* = 66/228), electrophysiological (*n* = 59), sound therapy (*n* = 56) and psychological (*n* = 47). The majority of trials evaluating electrophysiology-based interventions are restricted to a small number of candidates recruited to experimental research studies, and so this intervention strategy is not part of standard clinical practice. Hence, a decision was made to focus the Delphi process on sound-, psychology-, and pharmacology-based interventions to create three intervention-specific Core Outcome Domain Sets.

It is vital that all key stakeholders are involved in the development of a Core Outcome Domain Set to ensure that it has broad relevance. Patients and members of the public with lived experience of a condition are valued stakeholders in the development of relevant Core Outcome Sets because they have first-hand experience of that condition [10]. These groups are henceforth collectively referred to as ‘health care users’. Instances have arisen in previous Core Outcome Set work where health care users have identified outcome domains as important that were previously overlooked [11] or previously thought to be of little importance [12] (see also [13]). One of the attractive features of the Delphi process to health care users is the ability to provide anonymity to all respondents which can offset the shortcoming of conventional means of pooling opinions obtained from a group interaction where there can be power differentials between different stakeholder groups [10, 14, 15]. Nevertheless, Core Outcome Set developers have somewhat limited experience in engaging with health care users in the process of creating consensus [10, 16], and previous studies have reported low rates of recruitment [17]. The design of the present Delphi process incorporates several different methods for promoting the involvement and participation of health care users with lived experience of tinnitus to address the secondary research question concerning what methods work well for recruiting and retaining health care users, and what methods do not work so well.

In summary, further research work is urgently needed to advance the standards for clinical trials of interventions for tinnitus by developing an agreed list of outcome domains, distinct elements of tinnitus, that are critically important for determining whether a treatment has worked, using a process that promotes input from health care users and professionals with experience in tinnitus. This protocol defines the COMIT’ID study which uses a Delphi process and face-to-face meetings to establish consensus on which outcome domains should be measured when assessing the effect of sound-based, psychology-based, and pharmacology-based interventions in clinical trials for tinnitus.

### Aims

The primary aims of the COMIT’ID study are to:

1. Establish three Core Outcome Domain Sets, one for each of the main intervention strategies (sound-, psychology-, and pharmacology-based)
2. Identify the key outcome domains that are common across all three Delphi processes.

Two secondary aims are to:

3. Identify the strengths and limitations of the study design with respect to methods for recruiting and for reducing attrition of health care users
4. Investigate whether participation by professional experts in the Delphi process affects their subsequent use of the Core Outcome Domain Set, compared with those who do not participate.

## Methods

We will use the Delphi process to achieve a consensus of opinion from broadly representative and international expert stakeholder groups [15]. This is an observational study design comprising three independent Delphi processes, sponsored by the University of Nottingham and managed by the National Institute for Health Research (NIHR) Nottingham Biomedical Research Centre. A prospective study protocol was registered in the Core Outcome Measures in Effectiveness Trials (COMET) database [18]. This protocol describes version 2.0 (dated 14 March 2017), that was approved by the Proportionate Review Sub-committee of the West Midlands – Solihull Research Ethics Committee (REC reference 17/WM/0095, IRAS reference 220112) on 21 March 2017.

### Research steering group

A Research Steering Group has been appointed to oversee and manage the project. The group comprises international colleagues representing the identified intervention approaches (HFH, BM, AL), a patient and public involvement and engagement manager (AH) two health care users with lived experience of tinnitus, referred to as Public Research Partners (VC, BT), and the Study Management Team (KF, HS, DAH). The role of the Research Steering Group is to:

1. Support the development of the study protocol, specifically commenting on the feasibility of the Delphi process, reviewing study documentation (e.g. advertisements, Information Sheets, video instructions for the survey, website content) and to participate in a pilot of round 1 of the Delphi survey
2. Review the list of outcome domains and associated descriptions, specifically commenting on the readability of the outcome descriptions, the appropriateness of the grouping of outcomes into categories and providing any additional outcomes that they believe should be included in the first round.

### Eligibility criteria for the Delphi panels

A range of expertise within the panel is considered to be an important quality criterion for development of a Core Outcome Domain Set [19]. We will, therefore, seek to include representatives from four key stakeholder groups that may be particularly interested in managing chronic subjective tinnitus in adults. Specific inclusion criteria have been defined for each stakeholder group:

1. *Health care users* must have the experience of living with tinnitus for 3 months or more and have had experience with at least one sound-, psychology-, or pharmacology-based intervention for their tinnitus, or have the intention of undergoing that treatment in the future
2. *Health care practitioners* must have a clinical qualification, be currently employed by a public or private institution that provides a tinnitus service to patients, have specific experience in assessing, diagnosing or managing chronic subjective tinnitus in adults who receive, or have received, a sound-, psychology-, or pharmacology-based intervention.
3. *Clinical researchers* must have an academic qualification, be currently employed by a research organisation, have current or ‘recent-past’ experience in clinical research with studies that focus on questions of clinical efficacy (benefit) of a sound-, psychology-, or pharmacology-based tinnitus intervention in humans (i.e. a co-author on a relevant peer-reviewed journal publication in the past 3 years)
4. *Commercial representatives* must be currently employed by a company that develops, manufactures or sells relevant sound-, or pharmacology-based product(s) that may be used for alleviating tinnitus. *Funders* must be currently contracted by an organisation that has funded tinnitus research projects addressing sound-, psychology-, or pharmacology-based interventions in the last 3 years

General eligibility for participation in the Delphi process includes men and women aged 18 years or older, with sufficient command of English to read, understand and independently complete the questionnaires. An online screening stage will ask all participants to self-certify that they ﻿are an ‘expert’ in at least one of the three tinnitus-intervention strategies.

All enrolled Delphi panellists will be eligible to register their interest in attending a 1-day consensus meeting. However, allocation of places will be limited to those respondents who complete all three rounds of the online survey. None of the Research Steering Group members will be allowed to vote on domains in the consensus meetings because this risks inadvertently introducing a power differential across participants, but they can enrol in the online Delphi surveys (with the exception of KF and HS who will have access to the dataset for all analyses). Each expert panellist is permitted to enrol for more than one Delphi process if they meet the eligibility criteria, but would be invited to attend only one consensus meeting.

### Panel size and justification

There is no agreed method to statistically calculate a sample size for online Delphi surveys or for consensus meetings and no criteria against which a sample size choice can be judged (e.g. [20, 21]). Some individual studies indicate that stakeholder groups of around 20 can provide stable results [21]; and this defines our minimum sample size. However, one of the key deciding factors is that panel membership should adequately represent their corresponding stakeholder group (health care users, clinical researchers, etc.). Another key deciding factor is pragmatic; the global pool of experts meeting our eligibility criteria differs from one intervention to another. So it is not feasible for all three online Delphi surveys to have the equivalent sample size or target number of stakeholders.

Across the three Delphi processes, we aim to recruit a minimum target of 260 and an upper target of 420 experts spanning the range of stakeholder roles. Sound-based tinnitus interventions are the most accessible intervention in the UK [6] and devices and smartphone apps are widely available for self-management. Thus, a large pool of stakeholders is expected. Therefore, the intention is to enrol into the sound-based Delphi survey 60–90 health care users, 20–30 health care practitioners, 20–30 clinical researchers, and 20–30 commercial representatives and/or funders. For psychology-based tinnitus interventions, there are no specific commercial avenues or funding streams. Therefore, for this Delphi survey, we will enrol 40–60 health care users, 20–30 health care practitioners, and 20–30 clinical researchers. Pharmacological interventions are not widely accessible because there are no licensed drugs for tinnitus [22]. Instead, there are some established medications for comorbid symptoms such as insomnia, depression, and anxiety. Therefore, the number of professionals and members of the public with knowledge and experience of these interventions is expected to be more limited. For the pharmacology-based survey, the intention is to enrol 30–60 health care users, 10–20 health care practitioners, 10–20 clinical researchers, and 10–20 commercial representatives and/or funders. Experts will be allocated to the appropriate online survey based on their self-declared expertise.

The consensus meetings require in-depth discussions with smaller groups. Up to 20 expert panellists will be invited to participate in each of three face-to-face group consensus meetings (corresponding to the sound-, psychology-, or pharmacology-based online Delphi surveys). Enrolment will be balanced across stakeholder groups, where possible.

### Recruitment methods

Taking a business-informed approach has been shown to be beneficial to recruitment in several multicentre clinical trials [23]. Adopting an explicit marketing plan, engaging charities or participants to act as champions, delivering effective messages to multiple audiences at multiple levels and achieving clinician and public buy-in are all known effective ‘business’ comp-style components [24]. We are using a purposive sampling approach to recruit health care users from both clinical and non-clinical settings, and to recruit professional experts who maximise the international relevance of the study findings (see [13]). We have created a study recruitment plan with adequate resource allocation at the outset. Recruitment strategies include a number of planned methods approved by the Research Ethics Committee, some of which will be general and others will be targeted at particular stakeholder audiences. General e-promotion routes include a study webpage (http://www.hearing.nihr.ac.uk/research/outcome-measures-for-clinical-trials), a ‘research news’ feature on the health care user community website (https://www.tinnitustalk.com/), and regular updates on study progress via social media channels such as Twitter (e.g. @COMITIDStudy) and Facebook (e.g. hearingnihr).

Health care users will be targeted using several different planned recruitment routes. UK ‘Tinnitus Awareness Week’ (6–12 February 2017) shortly preceded the study launch and provided a platform to inform patients about this study and the importance of accurately measuring tinnitus complaints. An introductory feature published in the British Tinnitus Association *Quiet* magazine highlighted the upcoming study and enabled people to register for the study [25]. In addition, health care users in the UK are to be targeted using a traditional NHS recruitment route with five audiology centres designated as Participant Identification Centres, in addition to the lead site in Nottingham. Sites will display study posters in the audiology clinic waiting room, and hand out Participant Information Sheets, as appropriate. A small number of health care professionals in Europe will do the same in their tinnitus clinics, dependent on gaining appropriate local regulatory approval. Specific e-promotion routes include several patient organisations that have agreed to support the project by publishing newsletter articles and announcements to their members (e.g. the British Tinnitus Association, Action on Hearing Loss, the Deutsche Tinnitus-Liga e.V., the German Tinnitus Foundation Charité, and Association France Acouphènes). And finally, the lead study site has a participant database containing email contacts for approximately 1000 health care users who have self-declared tinnitus.

To recruit health care professionals, a number of professional networks and organisations will also circulate invitations to their membership (British Society of Audiology, British Academy of Audiology, ENT and Audiology News, the TINNET network funded by an EU COST Action BM1306, the International Collegium of Rehabilitative Audiology, and the Pharmacological Interventions for Hearing Loss Working Group at the Hearing Centre of Excellence in the USA). Collectively, these networks reach out to tens of thousands of professionals across the globe, but the disadvantage is that the approach can be somewhat impersonal. Parallel routes for recruiting health care professionals will, therefore, involve personal invitation via email or face-to-face contact. The Study Management Team has created a list of potential participants with relevant expertise using manual searches of the following information sources: first and last authors of relevant conference proceedings in last 3 years (e.g. Tinnitus Research Initiative, International Tinnitus Seminar, British Tinnitus Association conference); corresponding authors for each clinical trial of tinnitus identified in our previous systematic review [6]; and all authors of systematic reviews of tinnitus (Cochrane or otherwise) published in the preceding 5 years. An email request for relevant staff nominations has also been sent to members of the Hearing Industry Research Consortium representing the hearing-aid sector.

We will also personally approach editors of scholarly journals in the fields of audiology and otology. They will not be considered as a separate stakeholder because this role tends to be secondary to one of the above professional occupations. But participants who are journal editors will be recorded, allowing for this subgroup of data to be examined for any notable response differences during the analysis phase.

### The Delphi survey

**Fig. 1:**
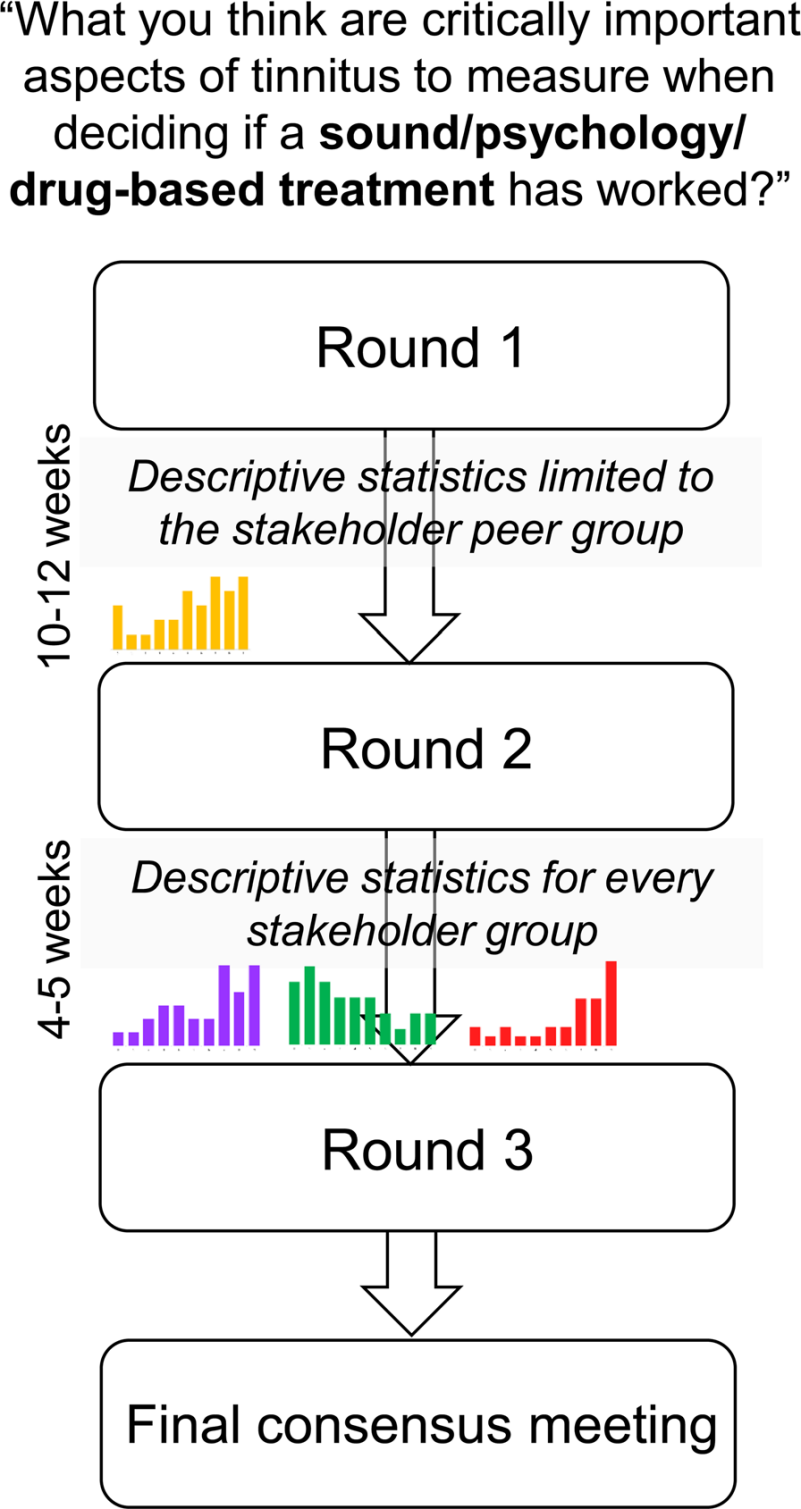
A schematic diagram of the Delphi process, including a time schedule of enrolment, online rounds 1–3, and the face-to-face consensus meetings. Only the initial question varies between the three surveys, as shown. The coloured histograms represent the planned graphical format of the results from the previous round. The single (yellow) histogram represents results for the peer stakeholder group. The Purple, green, and red histograms represent results for each relevant stakeholder group (peer and otherwise)

Three (parallel, independent) international Delphi surveys will be managed online using DelphiManager software maintained by the Core Outcome Measures in Effectiveness Trials (COMET) initiative (University of Liverpool: http://www.comet-initiative.org/). Each panellist will receive a Unique Identification Code and an e-link to the webpage for whichever survey matches their self-nominated expertise. Confirmation of eligible expertise will be collected online. A video explanation will illustrate how to complete the online tool. A flowchart of the study is shown in Fig. 1.

We will use a modified Delphi technique whereby we identify a ‘long-list’ of candidate outcomes prior to the first survey round, through systematic procedures that involve professional and public stakeholders and pool several independent sources of information [13, 26, 27]. For each outcome, we will provide the same plain-language description to participants in each stakeholder group. Each survey will comprise the same three sequential rounds, but will always be completed from the perspective of one of the interventions. All outcome domains will be retained from one round to the next presented in the same fixed order.

### The pre-Delphi stage involving health care users as Public Research Partners

We have completed the pre-Delphi stage and this created a finalised set of 66 outcome domains, each with a short plain-language description, grouped into 13 domain categories; the main content of the online Delphi surveys. Patient involvement has been an integral and transformative step of the study design process. To improve the appeal to health care users and to reduce attrition, two Public Research Partners on the Research Steering Group and one independent member of the public commented on the feasibility of the Delphi survey design, and reviewed study documentation (advertisements, Information Sheets, video instructions for the survey). In line with recommended considerations [10; 13], we also used qualitative research data gathered from health care users to make informed decisions about the main content of the online Delphi surveys and to ensure that outcome domain concepts are explained in ways that patient participants can understand and generate meanings which are consistent across all Delphi stakeholder groups. This goal is also highly relevant for any participants whose native language is not English; a factor for consideration in our international Delphi process.

**Fig. 2:**
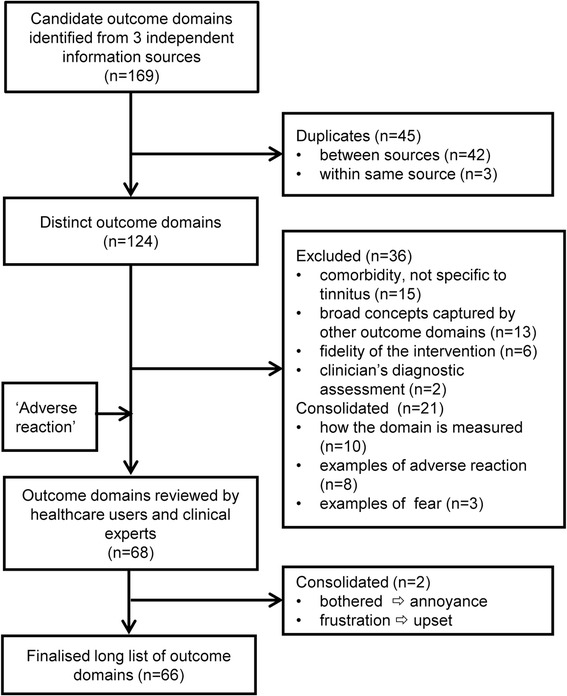
Flow diagram illustrating the pre-Delphi stage that has been completed with the involvement of health care users as Public Research Partners

The COMIT’ID Study Management Team scoped out information from three different sources. The first information source was a systematic review of outcome domains used in clinical trials of tinnitus treatments in adults which identified 62 outcome domains [6]. The second information source was an ongoing study that identified 64 outcome domains through thematic analysis of the items taken from 23 commonly reported tinnitus questionnaires [Fackrell, personal communication]. Both of these sources largely reflect outcomes that researchers have thought important to measure. The third source was a systematic review of dimensions of tinnitus-related complaints reported by patients and their significant others using questionnaire- and interview-based methods [28]. This source identified 43 outcome domains that reflected outcomes which patients considered important to them. This scoping process identified a total of 124 distinct outcome domains, after duplicates had been consolidated (see Fig. 2). For each outcome domain, associated examples were taken from each data source to aid interpretation of meaning for each outcome domain.

Our Research Steering Group, in particular our two Public Research Partners, informed our decision to reduce the long-list of outcome domains in order to improve the likelihood of completing all items in the Delphi survey. Discussion of each outcome domain and examples reduced the list of 124 by 57. Fifteen outcome domains were comorbidities that were associated with, but not specific to, tinnitus and so these can be considered out of the scope of this Core Outcome Domain Set and consequently were removed. Comorbidities were primarily related to hearing and mental health difficulties with examples including ‘hearing handicap’, ‘speech perception’, ‘reduced sound tolerance’, and ‘depression’.

Our Public Research Partners strongly argued that 13 outcome domains were broad concepts that were already encapsulated by other outcome domains in the list. Such hierarchies of constructs were considered a risk by confusing participants when rating the importance of individual outcome domains in the online Delphi survey. For example, ‘cognitive difficulties’ was already captured by ‘concentration’, ‘confusion’, ‘ability to ignore’ and ‘tinnitus-related thoughts’ and ‘health-related quality of life’ was already covered by ‘impact on relationships’, ‘impact on individual activities’, ‘impact on social life’, ‘impact on work’, and ‘sexual difficulties’. The Public Research Partners recommended that such broad concepts should be removed from the long-list and instead used as the category label.

Eight domains were not considered to be suitable as tinnitus-specific outcomes; two because they were restricted to the clinician’s diagnostic assessment and six because they were highly specific to the fidelity of the intervention. Diagnostic items were ‘causes of tinnitus’ and ‘tinnitus duration’. Intervention items were ‘adequacy of blinding’, ‘credibility of sham intervention (control)’, ‘credibility of real intervention’, ‘needling sensation (acupuncture)’, ‘masking (sound therapy)’, and ‘therapeutic alliance’. It is interesting to note that all of these candidates originated from the two information sources which reflected what researchers have thought important to measure, mostly from the systematic review of clinical trials [6].

A number of outcome domains were judged to relate to specific ways in which an outcome domain could be measured and thus, on a conceptual/semantic level at least, to be broadly equivalent to one another. For example, an ‘active task to distract or cope with tinnitus’ and ‘purposefully protecting or reducing the chance of potential problems’ were both considered different ways to measure the same health construct ‘coping’. This recommendation resulted in ten outcome domains being consolidated in the final version of the long-list under existing outcome domains ‘tinnitus loudness’, ‘tinnitus quality’, ‘coping’, and the category ‘body structures and functions’. Three further outcome domains were consolidated with the existing outcome domain ‘fear’ because they were considered by the Public Research Partners to be overlapping examples of the same concept (i.e. ‘fear for health’, ‘fear for quality of life’, and ‘fear of tinnitus becoming worse’).

Finally, eight outcome domains were consolidated because they were specific examples of what could be considered as a form of adverse reaction (i.e. ‘drug safety and tolerability’, ‘safety’, ‘safety and tolerability’, ‘side effects’, ‘tolerability’, ‘headache’, ‘pain frequency’, and ‘pain intensity’). ‘Adverse reaction’ was, therefore, created as a new outcome domain term.

These health care user-led decisions established a revised long-list of 68 distinct outcome domains. The Research Steering Group next took careful steps to identify the appropriate language for use in the Delphi survey. The task of phrasing each outcome domain, writing plain language concept definitions, and phrasing the names for domain category groupings was an iterative procedure conducted over two half-day workshop sessions with members of the Study Management Team working closely with the two Public Research Partners. Resulting outcome domain terms, concept definitions and category labels were reviewed by 14 members of the British Tinnitus Association’s Users’ panel and five clinical experts who were members of the British Tinnitus Association Professional Advisory Committee, and all members of the Research Steering Group for face validity, understanding, and acceptability.

At this stage, two further revisions were made to the long-list, arising from both health care user-led and professional-led recommendations about semantic equivalence. The first was that the outcome domain ‘bothered’, defined as ‘being disturbed by tinnitus or finding it a nuisance’, was consolidated with ‘annoyance’. The second was that the outcome domain ‘frustration’, defined as ‘feeling unable to change or achieve something because of or in relation to tinnitus’, was felt to be already represented by ‘annoyance’ and ‘upset’ and, therefore, was consolidated with these. Additional file 1 reports the finalised set of 13 domain categories, 66 outcome domains, and short plain language descriptions. Categories and outcome domains will be presented within the Delphi surveys in alphabetical order to avoid potential weighting [26].

### Round 1

For each outcome domain, participants will be asked to think about the importance of each tinnitus outcome domain and indicate how important it is to measure when deciding if a sound-based/psychology-based/pharmacology-based tinnitus treatment is working. Participants will be asked to score each outcome domain using the GRADE scale of 1–9, where 1 represents least important and 9 represents most important [29]. Selecting response options 1–3 indicates that the domain is ‘not important’, whilst 7–9 indicates that the domain is ‘critically important’ in deciding whether a tinnitus treatment is effective. Scores 4, 5, and 6 indicate that the outcome domain is ‘important but not critical’. If a participant feels that they did not understand a particular outcome, they will be able to select ‘unable to score’. Following each outcome and at the end of the round, optional open-text boxes will enable participants to add any comments. In round 1, participants will be able to propose additional outcome domains. These additional outcome domains will be reviewed and coded by two Study Management Team members, with appropriate plain-language concept definitions, to ensure that they represent new items for inclusion in rounds 2 and 3. Where uncertainty exists the Research Steering Group will be consulted, and all new outcome domain terms, concept definitions and category labels will be reviewed.

### Rounds 2 and 3

Participants will be eligible to continue to rounds 2 and 3 if they have responded to at least 40% of the outcome domains in the previous round. Corresponding data from those participants who responded to less than 40% will be removed. In rounds 2 and 3, all participants will receive the same list of outcomes with feedback tailored according to their Delphi allocation. The purpose of round 2 will be to enable participants to reflect on their scores in light of the viewpoint of their stakeholder peers, and to score the outcomes again. The purpose of round 3 will be to enable participants to reflect on their scores in light of the viewpoint of their stakeholder peers and all other stakeholder groups, and to score the outcomes again. Results will be presented graphically as well as numerically to improve visual appeal (Fig. 2).

After round 3, a short questionnaire with open- and closed-text response options will be emailed to all participants to collect feedback on their experience of being a participant, in addition to their perceptions of our strategies for recruiting and for reducing attrition of professional and health care users’ stakeholders.

### Consensus meeting

Professionals and health care users who have completed all three rounds of the Delphi survey and responded to at least 90% of the outcome domains in round 3 will be eligible to participate in the consensus meeting. As far as possible, allocated places will maintain the balance across stakeholder groups (e.g. 50% health care users/50% professionals) and will include non-UK, non-native, English language speakers. The purpose will be to agree a final Core Outcome Domain Set for each intervention category. Separate discussion within each meeting will include anonymised voting on each outcome as either ‘In’ or ‘Out’ (e.g. using electronic keypads which will create histograms and descriptive statistics ‘live’, to be displayed in the meeting).

### Consensus criteria

Consensus recommendations for each intervention category will be guided by the respective round-3 results. We will include domains for which 70% or more of the participants in each stakeholder groups score 7–9, and fewer than 15% score 1–3 [30]. We will exclude domains for which 70% or more of the participants in each stakeholder groups score 1–3, and fewer than 15% score 7–9 [30]. These decisions will be taken without further discussion because time at the consensus meeting will be limited. At the consensus meeting, the moderated discussion, voting and decisions will be guided as follows:

- For outcomes recommended to be included based on the round-3 analysis (70% scored 7–9), domains will be included if at least 70% of participants vote ‘In’
- For outcomes where at least 50% of more than one stakeholder group scored 7–9 on the round-3 analysis, domains will be included if at least 70% of participants vote ‘In’
- For outcomes where less than 50% of the participants in all stakeholder groups or at least 50% of only one stakeholder group scored 7–9 on the round-3 analysis, domains will be included only if at least 70% of participants vote ‘In’

If consensus is not reached after two rounds of voting, a ‘majority rules’ approach will be applied.

In the very final step, the Study Management Team will seek to establish a common cross-cutting Core Outcome Domain Set by identifying those outcome domains that were voted for inclusion in all three intervention-specific Core Outcome Domain Sets.

### Observational cohort study

We aspire to conduct a prospective observational cohort study to determine if participation of clinical researchers in the Delphi process increases uptake of the recommended Core Outcome Domain Sets for trials of sound-, psychology-, and pharmacology-based interventions for chronic subjective tinnitus in adults, compared with researchers who did not participate. This investigation will be conducted on the basis of the implementation of the recommendations in published reports of clinical trials in the 7 years after the Core Outcome Domain Sets are published.

### Analyses

The data from each Delphi round will be subjected to descriptive statistics, such as number completing each round, gender, country, region, native English language speaker (or not), and retention rate at rounds 2 and 3. We will plot the distribution of rating scores within each stakeholder group (including the ‘unable to score’ option and any missing data), and we will investigate attrition bias by comparing these plots across participants who complete successive rounds versus those who withdraw at rounds 2 or 3 [31]. We will also compare the distribution of rating scores for native and non-native English language speakers. Withdrawal and language are both factors that may affect how representative the final consensus might be of the target population. A separate analysis will assess the shifts in scores across rounds as a consequence of considering the anonymised feedback from other participants. The distribution of the scores for each outcome domain will be calculated for each stakeholder group within each intervention-specific Delphi survey.

The data from the feedback questionnaire will comprise open-text responses and these will be analysed using a thematic analysis approach to address one of the secondary objectives. Uptake of the recommendations will be analysed by calculating the number and proportion of authors who have implemented and referenced the COMIT’ID study recommendations in trials reported up to 7 years after their publication, comparing data between groups using non-parametric statistics (*p* < 0.05).

### Dissemination

Any important changes to eligibility criteria that are approved as a protocol modification will be communicated to our list of potential participants and advertised via all general e-promotion routes. Data from the final analyses of the Delphi surveys, consensus meetings and feedback questionnaire will be presented at relevant national and international conferences, including the British Tinnitus Association, the British Society of Audiology, the Tinnitus Research Initiative and the International Tinnitus Seminar. Several peer-reviewed publications are also planned; one addressing all primary objectives and one addressing the secondary objective for recruiting and for reducing attrition of health care users. Our findings will be further disseminated to members of the public and clinicians through specialist magazine articles, and our patient organisation supporters. Participants will not be identified in any publications.

## Discussion

This paper describes the design of a Delphi process to develop a Core Outcome Domain Set for sound-, psychology-, and pharmacology-based interventions for chronic subjective tinnitus in adults, to identify the strengths and limitations of the study design with respect to methods for recruiting and for reducing attrition of health care users across English-speaking and non-English-speaking countries, and to determine if participation increases adoption of the recommendations in the long term. We are not proposing that outcomes in a particular trial should be restricted to only those in the agreed list, but instead we expect that the Core Outcome Domains will be collected and reported in future trials, alongside additional outcomes that are deemed relevant. Results from the feedback survey can be used to inform the design of future consensus projects that seek to recruit and retain health care users.

To our knowledge, it is the first time that the Delphi technique incorporating these methods has been used for developing a Core Outcome Domain Set in tinnitus. It will supersede an earlier recommendation based on an informal 1-day workshop with 29 professional key opinion leaders [32]. Other important features of this study are that it will provide guidance for involving health care users in the development of the design and materials for the Delphi process. In addition to developing the Core Outcome Domain Set, this study may also shed light on controversial aspects within tinnitus trials, in particular differences in opinion across the three different intervention strategies concerning what is critically important for deciding whether or not the intervention has worked. The next steps in the process have been set out the COMiT roadmap [33]. These include a systematic appraisal of candidate instruments that adequately map onto the Core Outcome Domain Set and a statement about the preliminary Core Outcome Sets for sound-, psychology, and pharmacology-based interventions, with recommendations for further psychometric evaluation if required.

### Trial status

At the time of manuscript submission, all three Delphi surveys were open to recruitment.

## Additional file

Additional file 1: Table of domain categories, tinnitus-related outcome domains and concept definitions for all 66 outcome domains co-produced with health care users at the pre-Delphi stage. (XLSX 16 kb)

## Abbreviations

COMET: Core Outcome Measures in Effectiveness Trials
COMIT’ID: Core Outcome Measures in Tinnitus: International Delphi study
COST: European Cooperation in Science and Technology
ENT: Ear, nose and throat
NHS: National Health Service
TINNET: TINitus NETwork
UK: United Kingdom
USA: United States of America
